# A new species of the *Drawida japonica* species complex (Oligochaeta, Moniligastridae) disclosed by integrative taxonomy in Chongqing, China

**DOI:** 10.3897/zookeys.1278.187038

**Published:** 2026-04-30

**Authors:** SaiYu Qiao, Feng Zhang, Yaoguang Qin, Yufeng Zhang, Huifeng Zhao

**Affiliations:** 1 Hebei Key Laboratory of Zoological Systematics and Application, College of Life Sciences, Hebei University, Baoding, 071002, China Key Laboratory of Wetland Ecology and Environment, State Key Laboratory of Black Soils Conservation and Utilization, Northeast Institute of Geography and Agroecology, Chinese Academy of Sciences Changchun China; 2 Hebei Key Laboratory of Animal Diversity, Langfang Normal University, Langfang 065000, China Hebei Key Laboratory of Zoological Systematics and Application, College of Life Sciences, Hebei University Baoding China; 3 Key Laboratory of Wetland Ecology and Environment, State Key Laboratory of Black Soils Conservation and Utilization, Northeast Institute of Geography and Agroecology, Chinese Academy of Sciences, Changchun 130102, China Hebei Key Laboratory of Animal Diversity, Langfang Normal University Langfang China

**Keywords:** Earthworm, Integrative taxonomy, Oligochaeta, Southwest China

## Abstract

The *Drawida japonica* (Michaelsen, 1892) species complex is widely distributed across Asia, yet its taxonomy remains challenging due to morphological conservatism and cryptic diversity. Using an integrative taxonomic approach, a new species, *Drawida wuxiensis* Qiao & Zhao, **sp. nov**., is described from the Yintiaoling National Nature Reserve in Chongqing, China. The new species is characterized by its small body size (22.0–47.0 mm), the absence of dorsal and female pores, and a unique comma-shaped spermathecae distinguished from the similar species *D. japonica* sensu stricto, *D. henanensis* Liu & Zhao, 2025, and *D. sinensis* Liu & Zhao, 2025 within the same complex. Assemble Species by Automatic Partitioning (ASAP), the Generalized Mixed Yule Coalescent (GMYC) phylogenetic approach, and Bayesian Phylogenetics and Phylogeography (BPP) yielded congruent results recognizing the new species. Significant K2P genetic distances (18.0–19.9%) from related species also further corroborate its validity. Molecular phylogenetic analyses, incorporating 13 protein-coding genes from the mitochondrial genome and four nuclear loci, collectively support its stable basal phylogenetic position and further confirm its species status as an early-diverging lineage. This study revises the diversity profile of the *D. japonica* species complex and underscores the importance of integrating multiple lines of evidence for resolving species boundaries.

## Introduction

Earthworms, as key ecosystem engineers and sentinels, shape soil structure, and nutrient cycles, making their abundance vital for assessing soil health and environmental impacts (Bengrid et al. 2024; Li et al. 2024). However, earthworm taxonomy is limited by the scarcity of morphological characteristics and high homology (Marchán et al. 2022), especially for the family Moniligastridae Claus, 1880. Research on the Moniligastridae began with the description of the species *Moniligaster deshyaesi* Perrier, 1872. Since its description, the Moniligastridae has comprised 184 valid species across five genera (Liu et al. 2025a, 2025b; Narayanan et al. 2024a, 2024b, 2025a, 2025b). Among these genera, *Drawida* Michaelsen, 1900 is not only the most species-rich with 154 valid species and subspecies (Narayanan et al. 2024a, 2024b, 2025a, 2025b; Liu et al. 2025a, 2025b), but also widely distributed genus in Moniligastridae (Gates 1972; Jamieson 1977).

The taxonomy of *Drawida* is highly complex, which can be attributed to the following four major challenges. First, morphological characteristics exhibit considerable variation and are susceptible to interference. Morphological taxonomy relies on traits such as segment number, body color, and gizzard position, which show significant variation among individuals and species (Narayanan et al. 2024a). The clitellum in Moniligastridae consists of only a single layer of cells, making its boundaries difficult to define accurately, thereby posing significant challenges for species delineation (Blakemore et al. 2010, 2014). Furthermore, these morphological traits are significantly influenced by maturity, reproductive status, and specimen preservation methods (Liu et al. 2025b).

Second, synonymy is widespread. Due to the heavy reliance on morphological observations in early classifications, numerous species were repeatedly described or misclassified, leading to prominent issues of synonymy. For instance, *D. propatula* Gates, 1935 was initially distinguished by ovarian sac length. However, subsequent research confirmed that this trait varies with maturity, ultimately resulting in its classification as a junior synonym of *D. japonica* (Blakemore and Kupriyanova 2010). Similarly, *D. grahami* Gates, 1935 from China was also confirmed as a synonym of *D. japonica* due to minimal morphological differences (Blakemore et al. 2014).

Third, taxonomic criteria are limited and prone to misjudgment. Several key characteristics used in early studies, such as the positions of dorsal pores and atrium, were easily overlooked or misidentified. For example, the dorsal pores of *D. japonica* exhibit variability, being “discontinuous” or “completely absent.” In the Berlin syntype (ZMB Verm. 2122), they are restricted to intersegments 8/9, 10/11, and 31/32 and disappear entirely after segment 45, a discontinuity that was first documented by Blakemore et al. (2014) after re-examination of the type, but that had been overlooked by Michaelsen (1892) and subsequent authors such as Gates (1939). This omission resulted in incomplete species diagnoses. Further taxonomic confusion has been generated by inconsistent use of the term “atrium”, which several authors (Michaelsen 1900; Stephenson 1923; Gates 1972) have applied to either the spermathecal opening or the male pore (Blakemore et al. 2014).

Fourth, type specimens are missing. Many type specimens of early-described species are now lost or in poor condition, leading to ambiguity in taxonomic benchmarks. For example, the types of *D. japonica*, first described by Michaelsen (1892) based on specimens from Japan lacking specific type localities, are possibly lost now. Blakemore et al. (2014) mentioned a syntype that deposited in the Hamburg Museum with ID 403 (originally stored in the Zoological Museum, Berlin, with ID ZMB Verm. 2122). For the 30 type specimens of *D. changbaishanensis* Wu & Sun, 1996, that type locality is unknown (Wu and Sun 1996), and the specimens are already missing (HZ, pers. obs.). Additionally, the type specimens of *D. willsi* Michaelsen, 1907 and *D. calebi* Gates, 1945 are lost, making re-identification impossible (Blakemore and Kupriyanova 2010).

In *Drawida*, *D. japonica* is considered as peregrine, and the distribution area of *D. japonica* encompasses south, southeast, and east Asia (Gates 1936, 1972; Easton 1981; Blakemore 2012; Blakemore et al. 2014; Anderson et al. 2017; Narayanan et al. 2017, 2023, 2025a; Zhang et al. 2020). Blakemore et al. (2014) proposed that *D. japonica* should be regarded as a species complex since molecular evidence revealed substantial genetic divergence among specimens previously identified as this species. Liu et al. (2025b) accepted the classification of *D. japonica* species complex (DjSC), with delimitation of two species of *D. sinensis* Liu & Zhao, 2025 and *D. henanensis* Liu & Zhao, 2025.

At present, DjSC comprise *D. japonica* sensu stricto (s. s.), *D. sinensis*, *D. henanensis*, *D. calebi* Gates, 1945; *D. koreana* Kobayashi, 1938; *D. siemsseni* Michaelsen, 1910; *D. jeholensis* Kobayashi, 1940; *D. moriokaensis* Ohfuchi, 1938, *D. keikiensis* Kobayashi, 1938, and *D. companio* Blakemore, 2014 (Stephenson 1923; Kobayashi 1940; Blakemore et al. 2014; Narayanan et al. 2024a), a group of species sharing small body size (22–47 mm), grey or dark blue body color, the identical location of the male pore in 7/8 and spermathecal pore in 10/11 (Blakemore et al. 2014). The high degree of morphological similarity among species within the complex necessitates an integrative approach, combining meticulous morphological comparison with molecular evidence, for robust species delimitation.

Mitochondrial DNA (mtDNA) remains a prevalent molecular marker in earthworm research due to its ease of amplification, high evolutionary rate, and low recombination (Hurst and Jiggins 2005). However, it is susceptibility to lineage-sorting errors and limited representation of evolutionary history (Moritz et al. 1987). In contrast, nuclear locus, with biparental inheritance and deeper coalescence times (Palumbi et al. 2001; Hudson and Turelli 2003), offer robustness against sex-biased dispersal and enable the detection of hybridization and historical gene flow. These advantages establish nuclear markers as increasingly preferred tools in molecular species delimitation (Zink and Barrowclough 2008; James and Davidson 2012; Liu et al. 2025a, 2025b). Recently, more and more nuclear data have been provided in earthworm research (Zhang et al. 2012; Shen et al. 2018; Liu et al. 2025a, 2025b).

This study adopts an integrative taxonomic framework to delimit a putative new species, *D. wuxiensis* sp. nov., within the DjSC from southwestern China, combining morphological evidence with multi-locus data. Our molecular dataset includes the mitochondrial COI gene and three nuclear markers: 28S ribosomal RNA gene (28S), A-kinase anchor protein 17A (AKAP17), and flavin adenine dinucleotide synthetase 1 (FLAD1). Species boundaries were assessed by integrating morphological and several molecular delimitation methods, namely the Assemble Species by Automatic Partitioning (ASAP), the Generalized Mixed Yule Coalescent (GMYC), and Bayesian Phylogenetics and Phylogeography (BPP). Also, the mitogenomic data of the new species was used to perform its phylogenetic location in *Drawida*.

## Materials and methods

### Samples

The specimens were collected from Yintiaoling National Nature Reserve, Wuxi County, Chongqing Municipality, China (31.5164°N, 109.7331°E) by digging and hand-sorting (Table 1). The collected earthworms were stored in 95% alcohol in field work and preserved in -20 °C in laboratory in Langfang Normal University, Hebei, China (C-HLU).

**Table 1.** List of *Drawida* species incorporated in the analysis.

Specimen ID	Species	Location	Lat-Lon	Accession Number				
			N, E	COI	28S	AKAP17	FLAD1	Mitogenome
CQWX11_21	*D. wuxiensis* sp. nov.	China: Chongqing: Wuxi: Yintiaoling	31.5164, 109.7331	PX552882	–	–	–	–
CQWX11_29	*D. wuxiensis* sp. nov.	China: Chongqing: Wuxi: Yintiaoling	31.5164, 109.7331,	PX552882	–	–	–	–
CQWX11_38	*D. wuxiensis* sp. nov.	China: Chongqing: Wuxi: Yintiaoling	31.5164, 109.7331	PX552884	–	–	–	–
CQWX11_39	*D. wuxiensis* sp. nov.	China: Chongqing: Wuxi: Yintiaoling	31.5164, 109.7331	PX552885	–	–	–	–
CQWX11_44	*D. wuxiensis* sp. nov.	China: Chongqing: Wuxi: Yintiaoling	31.5164, 109.7331	PX552886	–	–	–	–
CQWX11_45	*D. wuxiensis* sp. nov.	China: Chongqing: Wuxi: Yintiaoling	31.5164, 109.7331	PX552887	PX558007	PX586779	–	PX369605
CQWX11_55	*D. wuxiensis* sp. nov.	China: Chongqing: Wuxi: Yintiaoling	31.5164, 109.7331	PX552888	PX558008	–	–	–
CQWX05_37	*D. wuxiensis* sp. nov.	China: Chongqing: Wuxi: Yintiaoling	31.4680, 109.8618	PX552881	PX558006	PX586778	PX586781	–
CQWX15_05	*D. wuxiensis* sp. nov.	China: Chongqing: Wuxi: Yintiaoling	31.5087, 109.8218,	PX552889	PX558009	PX586780	PX586782	–
CQWX15_06	*D. wuxiensis* sp. nov.	China: Chongqing: Wuxi: Yintiaoling	31.5087, 109.8218	PX552890	PX558010	–	–	–
CQWX15_22	*D. wuxiensis* sp. nov.	China: Chongqing: Wuxi: Yintiaoling	31.5087, 109.8218	PX552891	PX558011	–	–	–
HNLN_GR_I1_02	*D. henanensis*	China: Henan: Luoyang: Luonling	34.4364, 111.6368	PQ288546	PQ432438	–	–	PQ278996
HNLN_GR_I2_23	*D. henanensis*	China: Henan: Luoyang: Luonling	34.4364, 111.6368,	PQ288549	PQ432440	–	–	PQ278997
HNLN_GR_I2_26	*D. henanensis*	China: Henan: Luoyang: Luonling	34.4364, 111.6368	PQ288552	PQ432439	–	–	–
HNLN_GR_I2_27	*D. henanensis*	China: Henan: Luoyang: Luonling	34.4364, 111.6368	PQ288553	PQ432436	–	–	–
HNLN_GR_I2_29	*D. henanensis*	China: Henan: Luoyang: Luonling	34.4364, 111.6368	PQ288555	PQ432437	PQ452816	PQ452825	–
HNLN_GR_I2_44	*D. henanensis*	China: Henan: Luoyang: Luonling	34.4364, 111.6368	PQ288562	–	–	–	–
HNLN_GR_I2_51	*D. henanensis*	China: Henan: Luoyang: Luonling	34.4364, 111.6368	PQ288569	PQ432444	–	–	–
HNLN_GR_I2_53	*D. henanensis*	China: Henan: Luoyang: Luonling	34.4364, 111.6368	PQ288571	PQ432445	–	–	–
HNLN_GR_I2_54	*D. henanensis*	China: Henan: Luoyang: Luonling	34.4364, 111.6368	PQ288572	PQ432446	–	–	–
HNLN_GR_I3_29	*D. henanensis*	China: Henan: Luoyang: Luonling	34.4364, 111.6368	PQ288581	PQ432449	–	–	–
HNLN_GR_I2_34	*D. sinensis*	China: Henan: Luoyang: Luonling	34.4364, 111.6368	PQ288559	PQ432441	PQ452815	PQ452828	PQ278998
HNLN_GR_I2_40	*D. sinensis*	China: Henan: Luoyang: Luonling	34.4364, 111.6368	PQ288561	PQ432443	PQ452813	PQ452830	PQ278999
HNLN_GR_I3_02	*D. japonica* s. s.	China: Henan: Luoyang: Luonling	34.4364, 111.6368	PQ288578	PQ432448	PQ452809	PQ452827	–
HNLN_GR_I2_36	*D. japonica* s. s.	China: Henan: Luoyang: Luonling	34.4364, 111.6368	PQ288560	PQ432442	PQ452814	PQ452829	–
HNLN_GR_I3_01	*D. japonica* s. s.	China: Henan: Luoyang: Luonling	34.4364, 111.6368	PQ288577	PQ432447	PQ452810	PQ452826	PQ279000
MN539609	*D. japonica* s. l.	–	–	–	–	–	–	MN539609
KM199288	*D. japonica* s. l.	–	–	–	–	–	–	KM199288
OL840312	*D. ghilarovi*	–	–	–	–	–	–	OL840312
OL840313	*D. ghilarovi*	–	–	–	–	–	–	OL840313
LFSF_003	*D. gisti*	China: Hebei: Langfang: Anci	39.5222, 116.6644	PQ675805	PQ675807	PQ683865	PQ683867	–
E07_01	*D. gisti*	China: Tianjin: Binhai	39.0800, 117.6963	PQ675804	PQ675808	PQ683864	PQ683866	–
DLS_35								PX369606
E6_10								PX369607
	*E. irregularis*							OP381032
	*M. anisodiverticulus*							PV591013

### DNA extraction, amplification, and sequencing

Genomic DNA was extracted from posterior tissues using the TIANGEN Genomic DNA Kit (Beijing, China). Before tissue excision, specimens were arranged, photographed, and measured for morphological comparison.

The mitochondrial COI and nuclear 28S genes were amplified by standard PCR, while the nuclear genes AKAP17 and FLAD1 were amplified using nested PCR. Each 25-μL PCR mixture contained 1 μL of DNA template and 24 μL of PCR master mix (17.25 μL ddH_2_O, 2.5 μL Easy Taq-Buffer, 0.25 μL Easy Taq Polymerase, 2.0 μL dNTPs, and 1 μL each of forward and reverse primers). Primer sequences are listed in Table 2.

**Table 2.** Primers used for PCR and sequencing in this study.

Maker	primer	Sequence (5’-3’)	Round	Source
COI	LCO1490	GGTCAACAAATCATAAAGATATTGG		Folmer et al. (1994)
	HCO2198	TAAACTTCAGGGTGACCAAAAAATCA		
	COIE	TATACTTCTGGGTGTCCGAAGAATCA		Bely and Wray (2004)
28S	28sF1	GAGTACGTGAAACCGTCTAG		Pérez-Losada et al. (2009)
	28SR1	CGTTTCGTCCCCAAGGCCTC		
AKAP17	AKAP17-F1	AAYTGGGARGTNATGGARAA	Round1	Liu et al. 2025a
	AKAP17-R1	TCYTTRAACATNARYTTCAT		
	AKAP17-F2	AARATGATHAARCCNGAYCARTT	Round2	
	AKAP17-R2	GCYTTNACRAANCCCATRTAYTC		
FLAD1	FLAD1-F1	GGNCCNACNCAYGAYGAYAT	Round1	
	FLDA1-R1	TTNGGRTGNGTRTTYTCCAT		
	FLDA1-F2	TGYAARGCNTTYTTYGGNACNGA	Round2	
	FLAD1-R2	TTNACNCKCATRAAYTCNGGCCA		

The PCR protocol for COI consisted of initial denaturation at 95 °C for 5 min; 5 cycles of 95 °C for 30 s, 45 °C for 30 s, 72 °C for 50 s; 35 cycles of 95 °C for 30 s, 51 °C for 40 s, 72 °C for 50 s; and final extension at 72 °C for 10 min. For 28S: initial denaturation at 94 °C for 2 min; 35 cycles of 94 °C for 30 s, 50 °C for 40 s, 72 °C for 1 min 30 s; and final extension at 72 °C for 10 min. For AKAP17 and FLAD1, a two-round nested PCR was performed under identical conditions: 95 °C for 5 min; 35 cycles of 95 °C for 30 s, 45 °C for 30 s, 72 °C for 1 min; and 72 °C for 5 min.

PCR products were verified by 1% agarose gel electrophoresis and sent to Tianyi Huiyuan Biotechnology Co., Ltd. (Beijing, China) for Sanger sequencing on an ABI3730x platform. Sequences were assembled, aligned, and edited in MEGA 5 (Tamura et al. 2011). Genetic distances based on COI were calculated using the Kimura two-parameter (K2P) model (Kimura 1980) (Table 3).

**Table 3.** The COI genetic distance of K2P of groups of DjSC (values in %, and indicate intra-species genetic distance in parentheses).

	Species	1	2	3	4	5	6	7	8
1	*D. wuxiensis* sp. nov.	(0–0.6)							
2	*D. henanensis*	18.7–19.9	(0–6.8)						
3	*D. sinensis*	18.2–19.2	15.8–18.9	(11.5)					
4	*D. japonica* s. s.	18.2–18.4	17.5–18.8	17.3–18.3	(0–0.2)				
5	*D. koreana*	18.0–18.5	17.0–18.8	14.8–15.4	8.7–11.4	(6.4)			
6	*D. beiganica*	16.9–17.4	18.1–18.9	15.8–19.0	19.1–19.6	17.2–18.4	(0–0.2)		
7	*D. dongyinica*	19.5–20.5	16.5–18.3	18.5–19.5	15.4–15.8	16.8–17.7	13.6–14.1	(0–1.3)	(0)

### High-throughput sequencing and mitochondrial genome (mitogenome) annotation

To test the phylogenetic position of *D. wuxiensis* sp. nov., the mitogenome of CQWX11_45 is obtained through high-throughput sequencing using a paired-end 150 bp strategy on the DNAseq platform at BGI Genomics (Wuhan, China). The subsequent filtration of clean data from the raw sequencing data was conducted in accordance with the procedures delineated by Zhao et al. (2022). The mitogenomic sequence was assembled using MitoZ v. 2.4 (Meng et al. 2019). The assembled mitochondrial genome exhibited a circular conformation (Fig. 3), which was verified using the circle_check.py script from the MitoZ toolkit to confirm its structural completeness. The circular whole mitogenomic sequence with its annotation information were subsequently submitted to GenBank (accession number PX369605, Table 1).

### Morphological species delimitation

External and internal morphology of 11 clitellate specimens (CQWX05_37, CQWX11_21, CQWX11_29, CQWX11_38, CQWX11_39, CQWX11_44, CQWX11_45, CQWX11_55, CQWX15_05, CQWX15_06, CQWX15_22) were examined under a ZEISS stereomicroscope; images were captured with ZEN 3.3 pro software. Body length and width were measured to the nearest 0.1 mm. The prostomium, male and female pores, and spermathecal pores were documented. Internal organs (spermathecae, testis sacs, gizzards) were dissected and illustrated for subsequent species-level comparisons. The generic diagnoses and taxonomic assignments follow Blakemore et al. (2014), Michaelsen (1892) and Xu and Xiao (2011). All measures are based on the materials that preserved in alcohol. The comparison of key morphological characteristics of the *D. japonica* species complex is shown in Table 4.

**Table 4.** Key morphological characteristics of the species *Drawida japonica* complex and other related species (NA: not available).

Characteristic	*D. wuxiensis* sp. nov.	*D. henanensis*	*D. sinensis*	*D. japonica* s.s.	*D. koreana*	*D. calebi*	*D. jeholensis*	*D. siemsseni*	*D. impertusa*	*D. keikiensis*	*D. companio*
Color	Grey	Grey	Grey	Grey/Pale	Dark blue	Unpigmented	Whitish grey	Buff	Blotchy olive	Yellow/grey	Dark bluish
Length	22–47	24–47	41.8–52	28–70	63–100	35–83	52–66	110	45–90	40–54	80+
Width	3–5.5	1.8–3	3.5–4	2–4.5	Up to 4	2–4.5	up to 3.5	2–4	2.5–3.5	up to 2.5	NA
Genital markings	7.8–13, unpaired	7–11, unpaired	7–11, un-paired	7–13, unpaired	7–12, irregular	7–13 variously	7–11	7–12, irregular	NA	None	8
Clitellum	10–13; light pink	10–13; light grey	10–13; grayish white	9, 10–13, ½14	10–13; pinkish	NA	9–14	10–13	9,10–13	10–13	10–13
Spermathecae ducts	Long coiled	Medium coiled	Tight coiled	Long coiled	Short less coiled	Long coiled	Convoluted	Long coiled	NA	Long coiled	Moderately long
Spermathecae Atrium	Small oval-like in 8	Long sac-like and small in 8	Long sac-like and small in 8	Thumb-like and large in 8	Short, sac-like in 7	Conical in 8	Large in 7/8	Present in 8	NA	Small oral in 7/8	Small
Form of male pores	Spindle-shaped on 10–11	Penis-like overhanging on 10/11	Raised on 10/11	Stubby flab on 10 near 10/11	Penis-like on 10 near 10/11	Short penis at 10/11	10/11	10/11	lip-shaped on 10/11	Penis in pouch in 10/11	10/11
Female pore	Absent	Absent	Absent	11/12; near b-line	12; in b-line	NA	12; in ab-line	NA	11/12; in b-line	11/12; in b-line	12; in b-line
Ovisacs	12–13, extend to 15, 16	12–20	12–15	11/12–16	12–18, seldom 22 /23	20	16–20	NA	13/14/15	10/11/12–16 or 22	14
Gizzard segments and position	3; 12–18	2 or 3; 11/12–13/14 /16	2; 11–13	2 or 3; 11/12–13/14	2 or 3; 12–14	2–4; 12–17	2 or 3; 11/12–13	6	3 or 4;13/14–16 or 15–17	3–4; 12/13–15	3; 12–14
Vas deferens	Slender and less coiled	Slender and less coiled	Long coiled	Coiled and twisted	Loosely twisted	Short coiled	Short	NA	Loops	Short coiled	Coiled
Prostate	irregular disc-shaped	Elliptical white and thick disc-shaped	Long white and thick	Club-shaped and erect	Thumb-shaped	Sessile and smooth	Small and rugose	NA	Flat, sessile, almost circular, and “furry” glandular	Small and short, but broad and warty on surface	Small and white

### Molecular species delimitation

GMYC, ASAP and BPP are used to conduct molecular species delimitation analysis. ASAP is a recently developed method for rapid species delimitation from single-locus sequences by hierarchical clustering of pairwise genetic distances (Puillandre et al. 2020). GMYC (Fujisawa and Barraclough 2013) uses an ultrametric gene tree to estimate the transition rate between speciation and coalescent processes, thereby identifying independently evolving lineages (Dellicour and Flot 2015).

For single-locus screening, the mitochondrial COI and nuclear 28S fragments were analyzed with both ASAP and GMYC. ASAP was run on Kimura-2-parameter (K2P) distances with Pmin = 0.001, Pmax = 0.1, steps = 10, X = 1.0 and 20 bins (Puillandre et al. 2012). Ultrametric trees COI and 28S for GMYC were inferred in BEAST v. 1.7.5 (Drummond et al. 2012) under a Yule speciation prior and a strict clock; consensus topology was summarized with TreeAnnotator. GMYC analysis was then performed with the splits v. 1.0-19 package in R (Ezard et al. 2017).

Multilocus validation was conducted with BPP v. 2.2 (Yang and Rannala 2010) using the four-locus dataset (COI, 28S, AKAP17 and FLAD1). BPP implements a Bayesian Markov chain Monte Carlo (MCMC) algorithm under the multispecies coalescent model to calculate posterior probabilities of competing species-delimitation scenarios (Yang 2002, 2015; Rannala and Yang 2003; Flouri et al. 2018). Two analyses were conducted in BPP v. 2.2 (Yang and Rannala 2010); A00 analysis is conducted first using a fixed species tree to estimate population size and divergence time, A10 analysis use algorithm 1 to conduct species delimitation within-model parameter posterior (population size parameters, *θ_s_*; species divergence time, *τ_s_*) generated by running A00. The population size parameters (*θ_s_*) were assigned the gamma prior G (3, 1000), with a mean of 3/1000 = 0.003. The divergence time at the root of the species tree (*τ_0_*) was assigned the G prior (2, 1000), with a mean of 0.002, while the other divergence time parameters were specified by the uniform Dirichlet distribution (Yang and Rannala 2010).

### Phylogenetic analyses

Phylogenetic analyses were conducted using two distinct datasets. The first was based on the 13 PCGs from the mitogenomes of *Drawida*, with two species of *Enchytraeidae irregularis* Vejdovsky, 1879 and *Mesenchytraeus anisodiverticulus* Chen, 2012 serving as the outgroup (Table 1), and the PCGs were extracted using the gbseqextractor_v2.py script from the MitoZ toolkit (Meng et al. 2019). The second analysis utilized a combined dataset of three nuclear markers (28S, AKAP17, and FLAD1) with the mitochondrial COI gene, with *Drawida gisti* designated as the outgroup. Maximum Likelihood (ML) and Bayesian Inference (BI) methods were employed to reconstruct phylogenetic trees from MAFFT aligned sequences (Rozewicki et al. 2019). Substitution models for BI for the both dataset were selected using jModelTest2 (Darriba et al. 2020) based on the Akaike Information Criterion (AIC): PCGs for GTR+I+G4; TPM1uf+I+G4 for COI, GTR+I+G4 for 28S, TPM3+G4 for AKAP17 and TPM2uf+I for FLAD1.

ML analysis was performed in RAxML 8.2.12 (Stamatakis 2014). The GTR+GAMMA model, a nucleotide substitution model suitable for analyzing DNA sequence data, was selected. The outgroup *D. gisti* was used to determine the root node position of the system tree, and perform 1,000 rapid bootstrap iterations (bootstrap values, BV) to assess the support level for each branch within the tree, only branches with BV ≥ 50 % are retained and displayed in the final topology, and BV < 50 % are considered un-robust and were omitted. BI analysis was conducted using MrBayes v.3.2.6 (Ronquist et al. 2012), employing Markov Chain Monte Carlo (MCMC) methods for Bayesian phylogenetic inference, and run in two million generations to ensure the average standard deviation of split frequencies is less than 0.01. The results of p-files of BI were examined in Tracer v. 1.7.5 (Rambaut et al. 2018), and effective sampling size (ESS) values larger than 200 were accepted. Posterior probabilities (PP) quantify the statistical confidence in nodes. Branches with PP ≥ 0.95 are shown as well-supported, while those with PP < 0.80 are considered un-robust and were omitted. All of the resulting trees were visualized and edited using FigTree v. 1.4.2.

## Results

### Taxonomy

**Family Moniligastridae Claus, 1880**

**Genus *Drawida* Michaelsen, 1900**

#### Drawida wuxiensis

Taxon classificationAnimaliaMoniligastridaMoniligastridae

Qiao & Zhao sp. nov.

3D22C3CB-6D78-5BDF-AD07-55FB0C3DC204

https://zoobank.org/3E978B15-01F6-4669-BB54-5901030A452D

Figs 1− 4

##### Material examined.

***Holotype***: • one clitellate (CQWX11_45) (COI accession number: PX552887), Yintiaoling Nature Reserve (31.51855°N, 109.72814°E, 1942.72 m elev.), Wuxi County, Chongqing Municipality, 2025-05-11, coll. Huifeng Zhao. ***Paratypes***: • six clitellates [CQWX11_21 (COI accession number: PX552882) CQWX11_29 (COI accession number: PX552883), CQWX11_38 (COI accession number: PX552884), CQWX11_39 (COI accession number: PX552885), CQWX11_44 (COI accession number: PX552886), CQWX11_55 (COI accession number: PX552888)], the six specimens share identical collection data with the holotype.

##### Additional specimens.

Four clitellates. CQWX05_37 (COI accession number: PX552881), Yintiao Ridge Nature Reserve (31.4681°N, 109.82181°E, 1244.22 m elev.) Wuxi County, Chongqing Municipality, 2025-05-09. CQWX15_05, CQWX15_06 (COI accession number: PX552889, PX552890, respectively) and CQWX15_22 (COI accession number: PX552891) Yintiao Ridge Nature Reserve (31.50869°N, 109.8618°E, 1178.47 m elev.), Wuxi County, Chongqing Municipality, 2025-05-14. All the specimens of the new species are deposited in the Hebei Key Laboratory of Animal Diversity, Langfang Normal University, Hebei, China (**C-HLU**).

##### Diagnosis.

Length 22.0–47.0 mm, diameter 3.0–5.5 mm, 67–120 segments; body grey or white, head pale pink, clitellum light-pink to pale-yellow in X–XIII. Prostomium prolobous; dorsal pores absent. Setae four pairs per segment, aa = bc, ab = cd. Genital markings unpaired, irregular, VI/VII–XIII, maximum 4–7 papillae in XIII. Spermathecae paired in VII–VIII, ampulla white with slightly yellowish, comma-shaped or crescent-shaped in VIII, with a long, curly, tightly coiled duct, and atrium small oval shaped in VIII. Male pores paired, 10/11, between b and c, spindle-shaped, superficial, no penis. Female pores absent. Hearts 4 pairs (VI–IX). Septa 5–8 thick, muscular. Gizzards three, greyish-white with faint golden-caramel tint, XII(XIII)–XVIII(XX). The testis sacs are asymmetrically paired and pale brown or white, extending posteriorly from IX–XIII, occupying two or three segments; vas deferens slender, loosely coiled in IX–X; prostate glands one pair, elliptical, white, irregular-discoid, tightly adherent to parietes in XI. Ovisacs paired, umbrella handle-shaped, pink-tinged, XII–XIII, some extend to XV and XVI. Ovarian chamber absent. Accessory glands round, stalkless, corresponding to external genital papillae.

##### Description.

**Figure 1.**
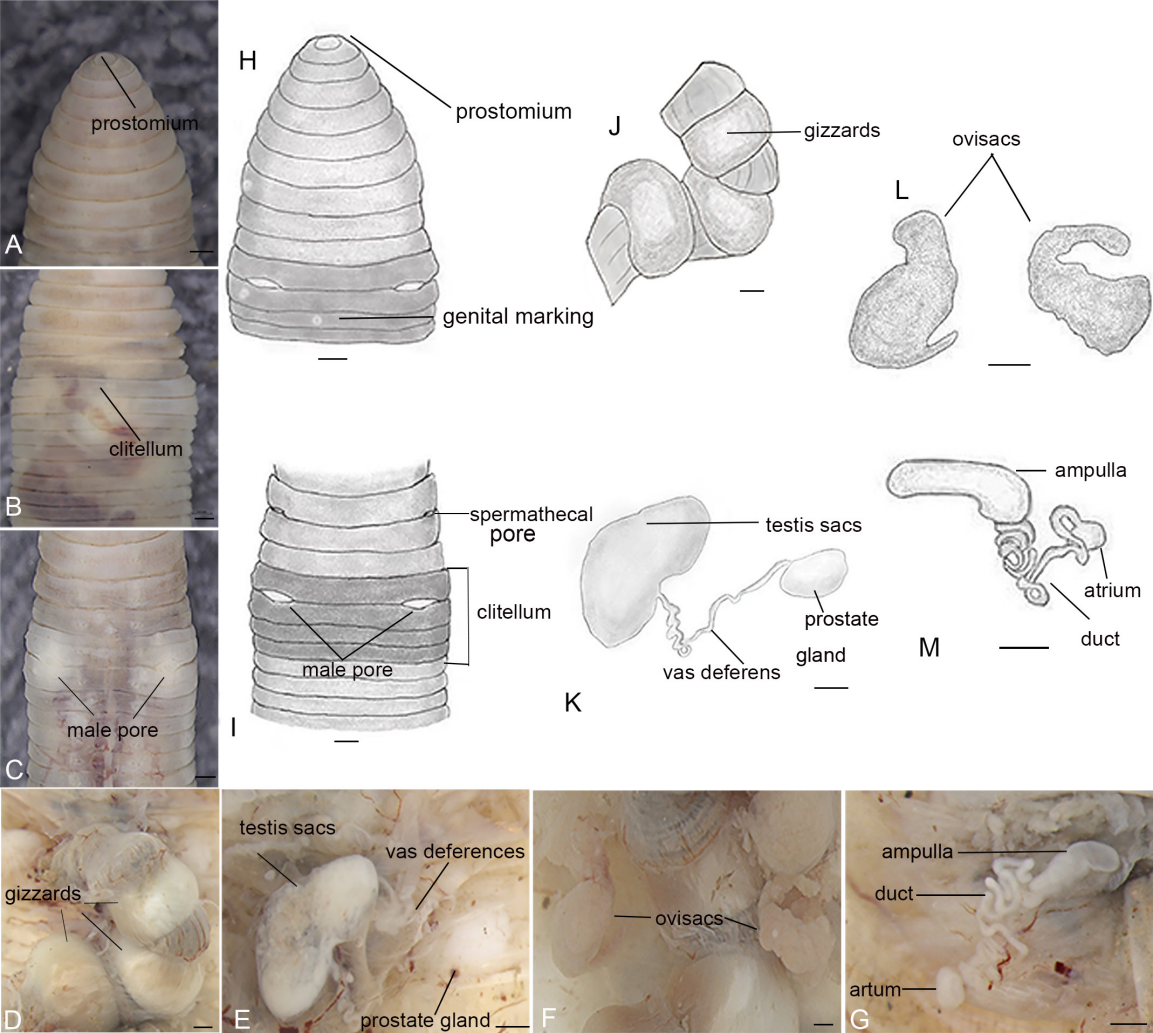
*Drawida wuxiensis* sp. nov., holotype (CQWX11_45). **A**. Ventral view of the prostomium and genital markings; **B**. Dorsal view of clitellum region; **C, I**. Ventral view of male pores, and spermathecal pores and clitellum region; **D, J**. Ventral view of gizzards; **E, K**. Right testis sac, with vas deferens and prostate gland; **F, M**. Ventral view of ovisacs (CQWX11_38); **G, M**. Right spermathecae. Scale bars: 0.5 mm.

***External characters***: Length 22.0–47.0 mm, diameter 3.0–5.5 mm, 67–120 segments. Head pale pink, body grey or white. Prostomium prolobous (Fig. 1A, H). Dorsal pores absent. Clitellum annular in X–XIII, light pink or pale yellow (Fig. 1B). Setae lumbricine, 4 pairs, small dots that exist in every segment except the first, about ab = cd, aa = bc. Genital markings present as small, white, papilla-like protuberances, unpaired irregularly in VI/VII–XIII. The highest number of papillae is typically observed in XIII of most specimens, with a range of 4–7. Spermathecal pore paired in 7/8 in bc-line, close to c. Male pores, paired in 10/11, between setae b and c, and the distances from b and c are approximately equal, spindle-shaped, superficial without protrusion or indentation, penis absent (Fig. 1C, I). Female pores absent.

***Internal characters***: Hearts four pairs in VI−IX. Septa V−VIII relatively thick and muscular, gizzards three in XII−XVIII or XIIII−XX segments, greyish-white with faint golden-caramel tints (Fig. 1D, J). The testis sacs are asymmetrical paired and pale brown or white, extend posteriorly from IX–XIII, occupying two or three segments, vas deferens slender and loosely coiled in IX−X, with one pair elliptical white and Irregular disc-shaped prostate gland tightly adhered to the abdominal wall in XI (Fig. 1E, K). Ovisacs paired, umbrella handle-shaped, tapering gradually toward the tip and pink-tinged, in XII−XIII, some extend to XV and XVI (Fig. 1F, L). Ovarian chamber absent. Spermathecae paired in VII−VIII, ampulla white with slightly yellowish, comma-shaped or crescent-shaped in VIII, with long, curly, and tightly coiled duct, atrium small oval shaped in VIII (Fig. 1G, M). Accessory glands round and stalkless, in correspondence with external genital markings.

##### Etymology.

The name refers to the type locality, Wuxi County in China.

##### Distribution.

Chongqing (China).

##### Habitat.

Grassland in high elevation in Chongqing Wuxi reserve.

##### Remarks.

DjSC currently includes *D. calebi*, *D. companio*, *D. henanensis*, *D. japonica* s. s., *D. jeholensis*, *D. keikiensis*, *D. koreana*, *D. moriokaensis*, *D. siemsseni*, *D. sinensis*, and the newly described species *D. wuxiensis* sp. nov. The new species exhibits a relatively short body length (22−47 mm), distinguishing it from *D. koreana*, *D. siemsseni*, *D. keikiensis*, *D. calebi*, *D. jeholensis*, *D. moriokaensis* and *D. companio* (Table 3). Additionally, *D. wuxiensis* sp. nov. displays a grey body color in alcohol, in contrast to the dark bule, buff and blotchy olive body color observed in *D. koreana*, *D. siemsseni* and *D. impertusa* (Hong 2002; Blakemore et al. 2014).

*Drawida wuxiensis* sp. nov. most closely resembles *D. henanensis*, *D. japonica* s. s. and *D. sinensis*, but differs from *D. henanensis* by having three gizzards (vs two or three in *D. henanensis*), and the position of gizzards range from XII–XVIII or XIV–XX (vs XI/XII–XIII/XIV/XVI), by the comma-shaped spermathecae with a small oral atrium and long coiled duct (vs the round ampulla with an elliptical atrium of *D. henanensis*); it can be distinguished from *D. japonica* s. s. by its shorter length (22−47 mm vs 28−70 mm), the absence of female pores (present in intersegment 11/12 in *D. japonica* s. s.), and gizzards occupying segments XII–XVIII/XIV–XX (vs XI/XII–XIII/XIV in *D. japonica* s. s.); the new species differs from *D. sinensis* by the comma-shaped ampulla and tightly coiled ducts (vs ball-like ampulla and loosely coiled duct of *D. sinensis*). Additionally, the new species bears paired, elliptical prostate glands whose volume equals one-quarter that of the testis sac, whereas the ratio is 1:3 in *D. henanensis*, 1:2 in *D. japonica* s. s. and 3:4 in *D. sinensis*.

### Molecular species delimitation

**Figure 2.**
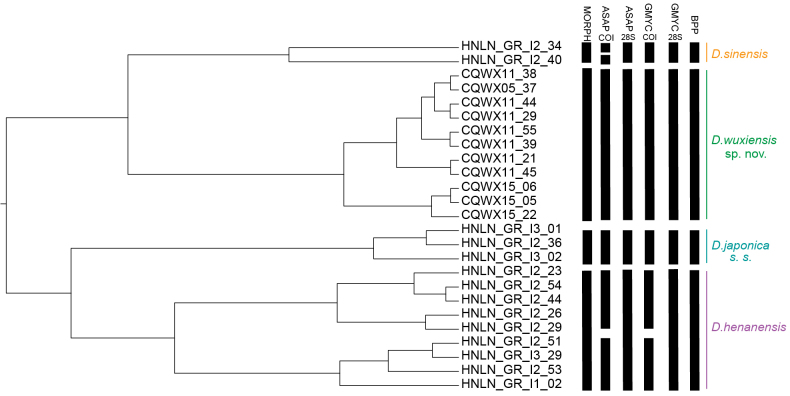
Species delimitation of *Drawida japonica* species complex. The bold bars show the specimen codes that were delimited by morphological characters and molecular data.

Three complementary methods: ASAP, GMYC and BPP were used to circumscribe molecular operational taxonomic units (MOTUs). BPP, applied to the four-locus dataset, consistently recovered the four morphologically defined species as distinct MOTUs. Single-locus analyses of 28S with both ASAP and GMYC produced congruent delimitations that matched morphology (Fig. 2). In contrast, COI-based analyses deviated from morphological boundaries: ASAP split *D. sinensis* into two MOTUs, whereas both ASAP and GMYC subdivided *D. henanensis* into two MOTUs (Fig. 2). K2P distances for COI underscored the genetic cohesion of the new species: intraspecific divergence was negligible (0–0.6 %), whereas divergence between *D. wuxiensis* sp. nov. and all other DjSC species ranged from 18.0 % to 19.9 % (Table 3).

### Phylogenetic relationship of DjSC

**Figure 3.**
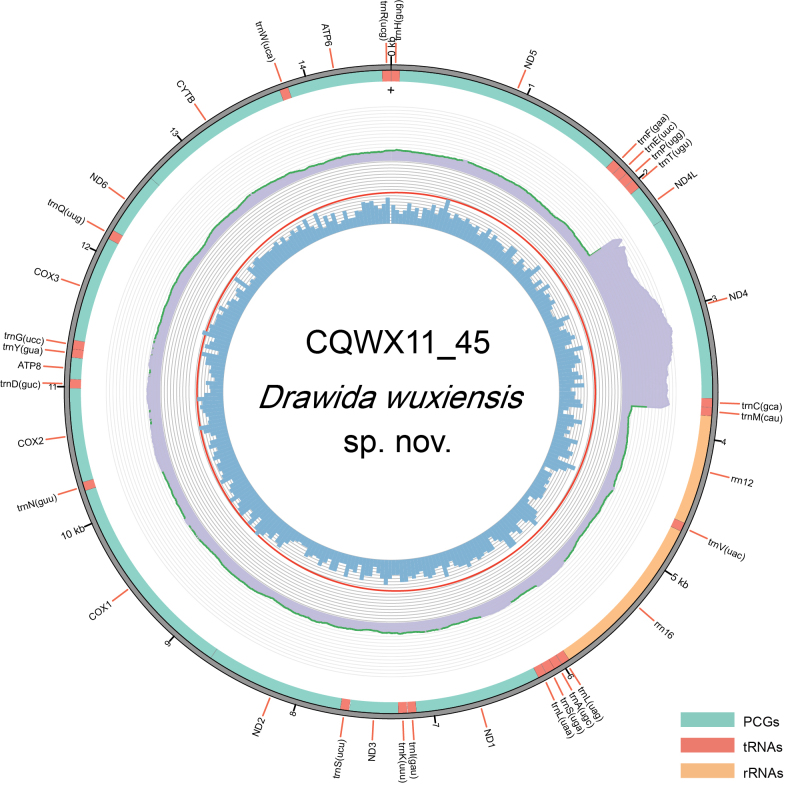
The circular mitogenome of *Drawida wuxiensis* sp. nov. The inner circle indicates the GC content, purple indicates the coverage across the mitogenome, and the outer circle shows the arrangement of the genes.

The mitogenome of *D. wuxiensis* sp. nov. is 14,617 bp long and contains the standard set of 37 genes: 13 protein-coding genes (PCGs), two ribosomal RNAs (rRNAs) and 22 transfer RNAs (tRNAs) (Fig. 3). Remarkably, it lacks the control region that is typically present in most earthworm mitogenomes (Zhang et al. 2016; Zhao et al. 2022).

**Figure 4.**
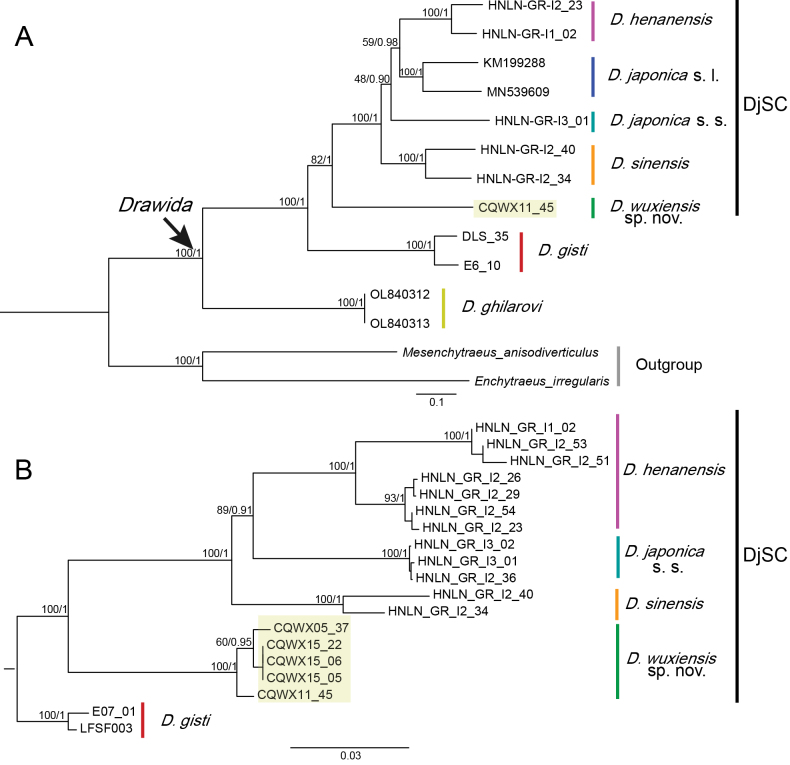
Phylogenetic trees of *Drawida japonica* species complex (DjSC) using the maximum likelihood method and Bayesian Inference. **A**. Based on the PCGs of the mitochondrial genomes; **B**. Based on a combination of COI, 28S, AKAP17, and FLAD1 genes. Numbers near branches indicate the maximum likelihood bootstrap support/Bayesian posterior probabilities. Bootstrap values and posterior probabilities are indicated on the tree. For clarity, values with a bootstrap value below 50 and a posterior probability lower than 80 have been omitted.

The phylogenetic tree of PCGs (Fig. 4A) illustrates the evolutionary relationships among the CDS genes of different species. It is evident that DjSC form a monophyletic group with robust support. Similarly, *D. henanensis* and *D. sinensis* also form a distinct cluster, showing their phylogenetic proximity. Additionally, *D. wuxiensis* sp. nov. appears as a separate branch, suggesting it may be a newly discovered species. The numbers at each node represent the support values for that branch, with higher numbers indicating greater confidence in the branch.

Phylogenetic trees constructed using ML and BI methods from polygenic site data encompassing COI, 28S, AKAP17, and FLAD1 genes (Fig. 4B) exhibited a topology highly similar to the ML tree and BI tree based on PCGs. Within this tree, the branch corresponding to DjSC received strong support, with both the posterior probability and confidence value reaching 100%.

## Discussion

An integrative taxonomic approach combining morphology and molecules supports the recognition of *D. wuxiensis* sp. nov. as a distinct member of the DjSC. The species is diagnosed by stable, unique characters of the male pores (position and shape), spermathecae (ampulla shape and duct coiling), seminal vesicles, ovaries, and prostates; detailed line drawings highlight these structures (Fig. 1). Compared with all congeners in the DjSC, *D. wuxiensis* sp. nov. differs consistently in body length, male-pore outline, and the degree of coiling of both the spermathecal duct and the vas deferens. These morphological disparities are matched by COI K2P divergences of 18.2–19.9%, well above the 15% inter-specific threshold routinely applied in earthworm taxonomy (Dong et al. 2019; Chang et al. 2024; Han et al. 2024, 2026; Qin et al. 2025; Zhao et al. 2025).

Phylogenetic analyses of the 13 PCGs showed *D. wuxiensis* sp. nov. at the base of the DjSC clade (Fig. 4), indicating that the new species represents an ancient, early-diverging lineage within the complex. Yintiaoling National Nature Reserve—the type locality of the new species—occupies a core sector of the Qinling–Daba biodiversity hotspot (Deng 2015). Rugged terrain and sharp elevational gradients (relief ca 2,400 m; mean elevation ca 1,900 m) have preserved the last intact primary forest in Chongqing Municipality. The species’ basal phylogenetic position and restriction to this single massif point to a deep history tied to the region’s geological past. Complex relief and pronounced climatic gradients create continuous microhabitat heterogeneity and act as topographic barriers that impede gene flow, forging a biogeographic refuge that fostered the survival and allopatric differentiation of *D. wuxiensis* sp. nov. Systematic field surveys across the Qinling–Daba ranges, coupled with population genomic dating, will refine divergence-time estimates and provide a full evolutionary portrait of this species.

This study delivers the first direct evidence that the “widespread” *D. japonica* sensu lato is a taxonomic chimera. *Drawida wuxiensis* sp. nov., is restricted to the Yintiaoling massif and incapable of long-distance dispersal, demonstrating that the alleged cosmopolitanism of *D. japonica* s. l. is an artefact of overlooked narrow-range endemics. Our integrative delimitation corroborates earlier hypotheses (Blakemore et al. 2014; Narayanan et al. 2024a, 2024b) and exposes a hidden radiation within the complex, nearly doubling the recognised species diversity in the DjSC. Continued application of genomic-morphological approaches is therefore essential to map the true distribution of each micro-endemic lineage and to stabilize the taxonomy of this iconic Asian earthworm group (Liu et al. 2025a, 2025b; Latif et al. 2025; Tiwari et al. 2025).

## Conclusions

Based on material collected from Yintiaoling, Wuxi County, Chongqing, we formally describe *D. wuxiensis* sp. nov. within the DjSC through an integrative taxonomic approach that combines fine-scale morphology with multi-locus phylogenetics. The new species confirms the extensive cryptic diversity hidden beneath the conservative external appearance of this complex and underscores Yintiaoling National Nature Reserve as both a cradle of differentiation and a critical refuge for the conservation of this enigmatic earthworm lineage.

## Supplementary Material

XML Treatment for Drawida wuxiensis

## Additional information

### Artificial Intelligence (AI) use

The authors accept full responsibility for the content of the manuscript, including the disclosure of any use of AI.

The following AI tools were used in the preparation of this manuscript:

**AI tools**: The large language model Kimi (developed by Moonshot AI).

**Used for**: Language, style and writing.

**Description**: The AI involvement was limited to language editing for clarity and grammar; no scientific content, data interpretation, or conclusions were altered.

### Author ORCIDs

S. Qiao https://orcid.org/0009-0002-9964-1352

F. Zhang https://orcid.org/0000-0002-3347-1031

Y. Qin https://orcid.org/0000-0001-8954-0182

Y. Zhang https://orcid.org/0000-0002-4066-6355

H. Zhao https://orcid.org/0000-0003-4243-9671

### Data availability

All of the data that support the findings of this study are available in the main text.
